# How do primary care clinicians approach hospital admission decisions for people in the final year of life? A systematic review and narrative synthesis

**DOI:** 10.1177/02692163241269671

**Published:** 2024-08-23

**Authors:** Rachel Davies, Matthew Booker, Jonathan Ives, Alyson Huntley

**Affiliations:** 1 Centre for Academic Primary Care, University of Bristol Medical School, Bristol, UK; 2 Centre for Ethics in Medicine, University of Bristol Medical School, Bristol, UK

**Keywords:** Hospitalisation, terminal care, clinical decision-making, primary health care

## Abstract

**Background:**

The final year of life is often associated with increasing health complexities and use of health services. This frequently includes admission to an acute hospital which may or may not convey overall benefit. This uncertainty makes decisions regarding admission complex for clinicians. There is evidence of much variation in approaches to admission.

**Aims:**

To explore how Primary Care clinicians approach hospitalisation decisions for people in the final year of life.

**Design:**

Systematic literature review and narrative synthesis.

**Data sources:**

We searched the following databases from inception to April 2023: CINAHL, Cochrane Library, Embase, MedLine, PsychInfo and Web of Science followed by reference and forward citation reviews of included records.

**Results:**

A total of 18 studies were included: 14 qualitative, 3 quantitative and 1 mixed methods study. As most of the results were qualitative, we performed a thematic analysis with narrative synthesis. Six key themes were identified: navigating the views of other stakeholders; clinician attributes; clinician interpretation of events; the perceived adequacy of the current setting and the alternatives; system factors and continuity of care.

**Conclusion:**

This review shows that a breadth of factors influence hospitalisation decisions. The views of other stakeholders take great importance but it is not clear how these views are, or should be, should be balanced. Clinician factors, such as experience with palliative care and clinical judgement, are also important. Future research should focus on how different aspects of the decision are balanced and to consider if, and how, this could be improved to optimise patient-centred outcomes and use of health resources.

**What is already known about the topic?**

- Hospital admissions towards the end-of-life carry significant risk of harm and may not be in keeping with a person’s wishes and goals of care.
- There is variation in rates of hospital admissions between geographical areas, services and clinicians.
- Interventions addressing system factors have thus far failed to improve rates of admission towards the end-of-life.

**What this paper adds?**

- This review focusses on the Primary Care clinician’s role in the hospitalisation decision.
- Decisions are complex and involve multiple stakeholders and factors.
- Navigating the views of other stakeholders is important and challenging.
- Factors specific to the clinician are important, including their experience of end-of-life care and clinical intuition.

**Implications for practice, theory or policy**

- The findings of this review suggest more work is needed to consider how clinicians can work with other stakeholders to reach consensus in decision-making.
- Resources and training could be used to improve clinician confidence in end-of-life care and making complex decisions involving other stakeholders.

## Introduction

During the final year of life many people experience an increase in the number and complexity of their health needs. Frequently this involves time spent in an acute hospital. A hospital admission during the final year of life can occur for many reasons ranging from the curative treatment of a new acute problem to managing complex symptoms during the final days of life. People in the final year of life are a heterogeneous group who may have any number of underlying pathologies such as cancers, organ failures, significant frailty or a combination of these. They do, however, have in common an underlying non-curable condition and the right to proactive person-centred care including crisis avoidance and the advance discussion of their wishes.^1^ The final year of life is substantially under-identified, making advance planning and coordination difficult.

Hospital admission decisions in the final year of life are important. Firstly, and potentially most importantly, because they can cause significant and long-lasting harms including loss of function, iatrogenic infection, falls and death in hospital where this would not have been a person’s wishes.^2,3^ Related to the risk of harms and death, an admission should form part of personalised care planning and decision-making for the last year of life, incorporating an individual’s goals and values, as well as a realistic and holistic assessment of the likely outcomes. Admissions towards the end-of-life may commonly be described as ‘inappropriate’ or ‘non-beneficial’, referring to aggressive or unnecessary treatments which don’t impact on the underlying chance of survival or quality of life.^4,5^ This description helps us to frame the problem but it does not necessarily help with proactive clinical decision-making- not least because there is no agreed definition as to exactly what ‘inappropriate’ or ‘non-beneficial’ look like.^6^ There is also no fixed conception of what a person’s wishes may be for their care in the final year of life with some suggestion in the literature that even an individual’s preference may change^7^ and should therefore be rediscussed often and in depth. It certainly is wrong to assume that no one in their final year of life should be admitted to hospital, it may frequently be necessary and/ or in keeping with their wishes.

The final reason for critiquing final year of life hospital admissions is economic: globally the population is aging and has increasingly complex health needs. In the UK, 81% of older adults have at least one admission in their last year of life^8^ with the cost of in-patient care significantly greater than community alternatives.^9^ No intervention to date has proved universally successful, and the significant variation in admission rates between services, places and times remains largely unexplained and context-specific.^10^

Here we focus specifically on the role of a primary care clinician in the decision-making process. The value of this comes from their wider role in providing community end-of-life care, and their ability to facilitate alternatives to admission. There is also evidence of inter-clinician variability, which is not well understood.^11^ By focussing on the primary care clinician role, it may also be possible to consider how policy or training could improve decision-making in this context.

## Clarification of concepts

### Final year of life

We aim to consider people who may be within the final 12 months of life. This is where many clinical guidelines suggest a threshold is crossed and where we should identify and offer additional supportive care for the end-of-life.^1^ We used a wide net to try and include all groups who may be within this category as these patients are often under-recognised, particularly those who may be in the final year of life due to frailty. Severely frail adults are five times more likely to die within a year than those who are not frail^12^ and they are also more vulnerable to post-hospitalisation harms.^13^ Severe frailty is also a state associated with significant uncertainty of trajectory, prognosis and outcomes which can make identification of the end-of-life and subsequent decision-making particularly challenging.^12,14,15^ There is evidence to suggest that up to 80% of residential home residents may be in their final year of life^1^ and thus this group have also been included.

We used database searches with broad range of terms to capture studies including patients who *may* be in the final year of life. Preliminary searching suggested that very few studies answering our question were using this term specifically. We did not want to limit our search to people who have been identified as ‘palliative’ or ‘end-of-life’ only as this would be likely to miss many people with the greatest uncertainty around prognosis and most challenging decision-making.

### Hospitalisation

Any move from a person’s usual place of care (including residential care) to a facility with higher acuity for any reason. We do not include transitions back to usual place of care or transfer to residential care from home within this discussion.

## Aim

To explore what is known about how Primary Care clinicians approach hospitalisation decisions for people in the final year of life.

## Methods

### Design

We performed a systematic review of published literature followed by a narrative synthesis of the findings as per Popay et al.^16^ The review protocol was prospectively registered with Prospero (CRD42022361547).

### Inclusion and exclusion

#### Inclusion and exclusion criteria

**Table:** 

Inclusion criteria	Exclusion criteria
Clinician population: primary care or community-based clinicians making hospitalisation decisions	Clinician population: all other clinicians making hospitalisation decisions
Patient population: adults who may be in the final year of life/who are frail/multimorbid and elderly	Patient population: people under 18 years, those who are not likely to be in the final year of life
Study characteristics: - Studies specifically considering the decision-making process. - Studies specifically considering the clinicians role in the decision-making process. - Qualitative or quantitative studies	Study characteristics: - Studies focussing on the role of others in the decision-making process.
There were no date restrictions	

### Data sources

Six databases were searched in April 2023: Medline, Embase, CINAHL, PsychINFO, Web of Science, Cochrane Library. Reference and forward citation reviews (via Google Scholar) were performed on the included records. Table 1 shows the full search strategy.

**Table 1.** Full search strategy from Medline.

Facet 1		Facet 2		Facet 3		Facet 4
Frail, elderly (MeSH)	*AND*	Hospitalisation (MeSH)	*AND*	Decision-making (MeSH)	*AND*	General Practice (MeSH)
Frail		Hospitali?ation		Clinical decision-making (MeSH)		General Practitioners (MeSH)
Elderly		Emergency admission		Decision?mak*		Family Practice (MeSH)
Aged, 80 years and over (MeSH)		Avoidable admission		Decision		GP
Multimorbidity (MeSH)		Hospital admission				General Practic*
Multiborbid*		Treatment escalation				Family Practic*
Terminal care (MeSH)						Primary Health Care (MeSH)
Palliative Care (MeSH)						Primary Care
Palliative						Doctor
						Nurse
						Community health nursing (MeSH)
						District nurs*

### Search strategy

References were downloaded into EndNote and duplicates were removed. Two of the authors (RD and AH) independently screened the titles, abstracts and full texts against the above inclusion and exclusion criteria.

### Quality assessment

Based on our knowledge of the field, we anticipated that the search would produce largely qualitative literature with some studies only partially addressing our question. We used the Critical Appraisal Skills Programme (CASP) checklist for qualitative research^17^ to assess whether broad quality indicators were met. We planned only to exclude records that were methodologically ‘fatally flawed’.^18^

### Data extraction and synthesis

The data extraction and synthesis were conducted by RD and checked by AH with frequent discussion with MB and JI. Our synthesis was guided by Popay’s guidance on performing a narrative synthesis within systematic reviews.^16^ We conducted a preliminary synthesis to identify the main/ most important concepts across the studies. Firstly, we identified individual themes using the authors’ own language, and their own definition of what was key or most important. We then combined these first themes across the studies based on our interpretation of their underlying meaning, to build relationships between the studies. These cross- study themes and sub-themes are shown in Figure 2.

## Results

**Figure 1.**
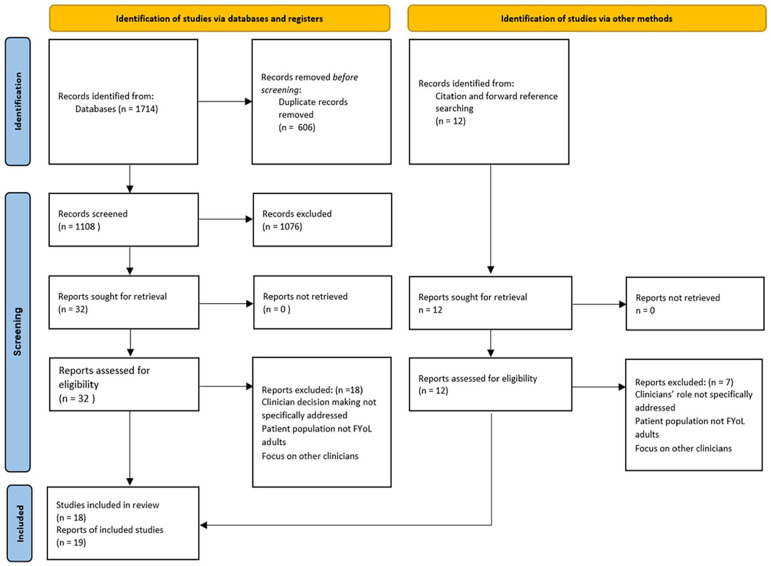
PRISMA flow chart.

The search returned 19 relevant records describing 18 distinct studies. Figure 1 shows the PRISMA flowchart of how these were identified.

A total of 14 of these studies were qualitative, 1 mixed method and 3 were quantitative. None were excluded based on quality.

### Study characteristics

Most included studies were qualitative (*n* = 14). Of these, 12 used interviews, 2 used focus groups and 1 used interviewing alongside observation. All the quantitative studies (*n* = 3) used questionnaire data, as did the mixed method study which involved a questionnaire with both open and closed questions. Table 2 shows all included studies.

**Table 2.** Included study characteristics.

Author, Year, Country	Title	Study type	Methods	Clinician population	Patient population	Clinical setting	Summary of findings
J. Cohen-Mansfield and S. Lipson, 2003, US	Medical staff’s decision-making process in the nursing home	Quantitative	Questionnaire	Nursing home physicians and ANPs	Nursing home residents (only without capacity)	Nursing home	Quantitative survey study with physicians and nurse practitioners. They were asked to rank the importance of different decision-making factors regarding ‘status change’ decisions of which hospitalisation was often an option (76% of cases). Reports importance of perceived quality of life, relative effectiveness of treatment options, families wishes.
J. Cohen-Mansfield and S. Lipson, 2006, US	To hospitalize or not to hospitalize? That is the question: An analysis of decision making in the nursing home	Mixed	Questionnaire with open questions	Nursing home physicians and ANPs	Nursing home residents (only without capacity)	Nursing home	A mixed methods study re-examining the same data set as used in Cohen Mansfield 2003, looking at only cases where a hospitalisation decision was made. Considers which factors are important in the decision. They found patient’s perceived quality of life and the standard practice for a condition to be the most significant factors. Includes some qualitative analysis of other factors from the open questions in the questionnaire.
A. Dreyer, R. Forde and P. Nortvedt, 2010, Norway	Life-prolonging treatment in nursing homes: How do physicians and nurses describe and justify their own practice?	Qualitative	Interviews	Nursing Home Physicians and nurses	Nursing home residents	Nursing home	Qualitative interview study with nursing home physicians and nurses on how they approach life prolonging treatments in general, in which hospitalisation is given as one. Findings include inadequate descriptions of competency assessment and therefore unlikely adequate inclusion of patient in the decision, the role of families in the decisions including occasions when families put pressure on clinicians to make a decision not in the interests of the patient. Overall concludes that better decision-making with patients and families is needed in order to better respect patient autonomy.
C. McDermott, R. Coppin, P. Little and G. Leydon, 2012, UK	Hospital admissions from nursing homes: a qualitative study of GP decision making	Qualitative	Interviews	GPs	Nursing home residents	Nursing home	Qualitative interview study with UK GPs investigating what factors influence their decision to admit frail NH residents to hospital. They conclude that the most important factors are clinical assessment, perceived risks and benefits to the admission and patient/family preferences.
T. Reyniers, D. Houttekier, H. R. Pasman, R. V. Stichele, J. Cohen and L. Deliens, 2014, Belgium	The family physician’s perceived role in preventing and guiding hospital admissions at the end of life: a focus group study	Qualitative	Focus groups	Family Physicians	Dying patients	Community	Qualitative study using focus groups to explore the views of Belgian FPs on making decisions for dying patients in the community. It reports the importance of advance care planning for acute decisions and the role of the FP being to mediate between the views of the other stakeholders but that in the Belgian context they may have less of a gatekeeper role and their views may be of secondary importance to those of patients and families.
T. Reyniers, D. Houttekier, J. Cohen, H. R. Pasman and L. Deliens, 2014, Belgium	What justifies a hospital admission at the end of life? A focus group study on perspectives of family physicians and nurses	Qualitative	Focus groups	Family Physicians and nurses	Dying patients	Community	This focus group study uses some of the same data set as the previous study but uses more of a retrospective lens to consider more objectively how admissions can be justified, rather than how individuals approach them. Main findings include that patient wishes to go to hospital can override almost all others with capacity of the current setting and some medical situations that also cannot be managed at home also being important.
M. Sercu, V. V. Renterghem, P. Pype, K. Aelbrecht, A. Derese and M. Deveugele, 2015, Belgium	‘It is not the fading candle that one expects’: general practitioners’ perspectives on life-preserving versus ‘letting go’ decision-making in end-of-life home care	Qualitative	Interviews	GPs	Dying patients	Community	This interview study with Belgian GPs focusses on their overall approach to making decisions for dying patients at home and whether they take life preserving or ‘letting go’ measures. Admission to hospital is included in life preserving measures but the specific aim is more in how the GPs perceive their overall treatment goals. Hospitalisation may be considered specifically after events of acute medical change, such as when situations become stressful.
M. Glogowska, R. Simmonds, S. McLachlan, H. Cramer, T. Sanders, R. Johnson, et al., 2016, UK	‘Sometimes we can’t fix things’: a qualitative study of health care professionals’ perceptions of end of life care for patients with heart failure	Qualitative	Interviews	GPs and community matron (also secondary care and speciality community services)	End stage heart failure	Community	This interview study with GPs, cardiologists and heart failure specialist clinicians explores issues around heart failure at the end-of-life including questions around hospitalisation decisions. Relevant conclusions are that in HF it can be hard to identify when people are dying and to access community PC support, and that hospitalisation remains the default option in cases of deterioration in HF.
T. Reyniers, L. Deliens, R. Pasman, R. Vander Stichele, B. Sijnave, D. Houttekier, et al., 2016, Belgium	Reasons for terminal hospital admissions: Results of a survey among family physicians	Qualitative	Interviews	Family physicians	People who later died in hospital whose death was not unexpected	Community	A questionnaire study with Belgian GPs exploring reasons behind hospitalisation for patients who had expectedly died there. They report perceptions of the care setting as inadequate, patient preferences and family belief that the hospital is a better place of care, and FPs familiarity with the patient and their wishes as the most significant factors.
R. Palan Lopez, S. L. Mitchell and J. L. Givens, 2017, US	Preventing Burdensome Transitions of Nursing Home Residents with Advanced Dementia: It’s More than Advance Directives	Qualitative	Interviews	Nursing home physicians	Nursing home residents with advanced dementia	Nursing homes	An interview study with NH physicians and nurses in the US, specifically looking at how decisions regarding admitting residents with advanced dementia are made. They conclude that the wishes of surrogates likely to be most important.
M. Hazelhoff, M. A. Pouw, G. A. Welker, J. J. Knol and S. E. J. A. de Rooij, 2018, Netherlands	Factors influencing professional decision making on acute hospital referral in the case of elderly patients with cognitive impairment among general practitioners (GPs) in The Netherlands: a qualitative study	Qualitative	Interviews	GPs	Elderly with cognitive impairment	Community	An interview study with Dutch GPs on how they perceive decisions on hospitalisation for elderly patients with cognitive impairment are made. They place great importance on the views of patients and families and the influence on decisions.
M. K. Glette, T. Kringeland, O. Roise and S. Wiig, 2018, Norway	Exploring physicians’ decision-making in hospital readmission processes – a comparative case study	Qualitative	Interviews and observation (comparative case study)	GPs and nursing home physicians	Elderly	Community and Nursing Homes	A comparative qualitative study looking at readmissions to hospital between two municipalities and the reasons for possible differences. Some results relating only to readmission situations but some relevant themes to admissions in general.
S. Amadoru, J.-A. Rayner, R. Joseph and P. Yates, 2018, Australia	Factors influencing decision-making processes for unwell residents in residential aged care: Hospital transfer or Residential InReach referral?	Qualitative	Interviews	GPs and Aged Residential Facility staff involved with decision-making	Aged residential facility residents	Aged Residential facilities	A qualitative interview study with Australian GPs and ARF staff (involved in clinical decision-making) specifically looking at how hospital vs residential in reach referrals are made. Important themes highlighted are patient and family preferences, availability of nursing and medical care and facility policies that might mandate hospital transfer.
S. Giezendanner, K. Bally, D. M. Haller, C. Jung, I. C. Otte, H. R. Banderet, 2018, Switzerland	Reasons for and Frequency of End-of-Life Hospital Admissions: General Practitioners’ Perspective on Reducing End-of-Life Hospital Referrals	Quantitative	Questionnaire	GPs	Palliative patients	Community	A quantitative survey study from Switzerland exploring GP opinions on EOL admissions. They conclude that the presence, adequacy and ability to continue of the informal care network is important, as is the complexity of symptom control, the wishes of the patient and family and the GPs confidence in palliative care competencies.
J. J. Chen, K. Gamble, L. Graham-Wisener, K. McGlade, J. Doherty, P. Donnelly, 2018, UK	GP perceptions of the adequacy of community-based care for patients with advanced heart failure in a UK region (NI): a qualitative study	Qualitative	Interviews	GPs	Patients with severe heart failure	Community	A qualitative interview study with GPs which covers the broader topic of adequacy of care for patients with significant HF. It does address reasons why they may be admitted to hospital, specifically that hospital is seen as the default option, that it will provide better care and the lack of availability of other options due to lack of community services and lack of specialist support/ communication.
S. Hoare, M. P. Kelly and S. Barclay, 2019, UK	Home care and end-of-life hospital admissions: a retrospective interview study in English primary and secondary care	Qualitative	Interviews	GPs and community nurses (also ambulance staff, secondary care staff and families)	Patients admitted to hospital near the end-of-life	Community	A qualitative interview study in the UK exploring why people being cared for at home are admitted to hospital for the end-of-life. It explores the views of GPs and community nurses but also emergency ambulance staff and secondary care clinicians. It focusses on the difficulties of accessing professional/nursing care and the difficulties for families in providing care: emotional, physical and the extend of required care.
A. Moore, C. Croxson, S. McKelvie, D. Lasserson and G. Hayward, 2019, UK	General practitioners’ attitudes and decision making regarding admission for older adults with infection: a UK qualitative interview study	Qualitative	Interviews	GPs	Older adults with infection	Community	A qualitive interview study with UK GPs exploring how they make admission decisions regarding older adults with infection. As well as clinical factors they explore risk, the emotional burden on GPs and the workload implications of their decisions.
V. Kouyoumdjian, E. Perceau-Chambard, C. Sisoix, M. Filbet and C. Tricou, 2019, France	Physician’s perception leading to the transfer of a dying nursing home resident to an emergency department: A French qualitative study	Qualitative	Interviews	Aged residential facility medical directors	Dying residents	Residential Facilities	A qualitative interview study with French medical directors. Includes patient, clinician and family factors. Acknowledges that some circumstances may be unique to France with the presence of the MD in NHs and the possible overlapping role with GPs.
E. S. Forsgarde, M. Roost, C. Elmqvist, B. Fridlund and A. Svensson, 2023, Sweden	Physicians’ experiences and actions in making complex level-of-care decisions during acute situations within older patients’ homes: a critical incident study	Qualitative	Interviews (critical incident technique)	Physicians	Older patients with acute medical situation	Community	Swedish qualitative study exploring how physicians make ‘level of care’ decisions for older patients with an acute medical change. Main findings are the importance of collaboration and understanding and guiding the wishes of other stakeholders.

Five studies were conducted in the UK, four in Belgium, two in the US, two in Norway and one each in Australia, France, Netherlands, Sweden and Switzerland. One study was in Dutch language which was machine translated by Google translate^19^ then checked and discussed by a native Dutch speaker with experience of the field. The remaining studies were in English. The clinical setting was split between patients in the community (*n* = 11) and residential care facilities (*n* = 6) with one study covering both.

Clinician participants included general/ family practitioners (*n* = 13), nursing home physicians (*n* = 5) and nurses in a decision-making role (*n* = 6). Seven studies included more than one of the professional groups. One study also included interviews with secondary care clinicians.^20^ Most participant numbers were small.

The patient groups were nursing home/ residential care residents without further specification (*n* = 4), nursing home residents who were dying (*n* = 1), nursing home residents with cognitive impairment/ without capacity (*n* = 2), people at the end-of-life at home (*n* = 4), people who had been admitted to hospital at the end-of-life (*n* = 2), older adults (*n* = 3), older adults with cognitive impairment (*n* = 1) and people at home with end stage heart failure (*n* = 2). One study included both community and nursing home residing older adults.^21^

A total of 12 of the studies focus specifically on hospitalisation decisions while 6 look at ‘level of care’ or treatment escalation decisions in which hospitalisation features as an option.

### Themes

**Figure 2.**
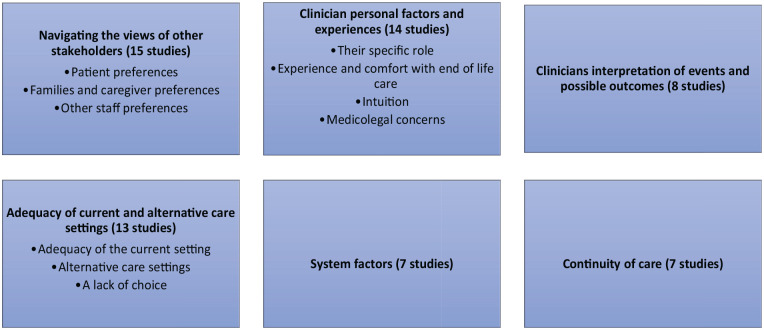
Themes and subthemes.

We identified six main themes across the studies with three of these broken down into subthemes, displayed in Figure 2, with the number of studies contributing.

#### Navigating the views and input of other stakeholders

The collaboration with other stakeholders and the influence that this has on the clinicians’ decision-making is discussed by all the studies. Overall, the views of the patient and their family/ caregivers’ views are given the most importance. This theme can be divided into patient preferences, family preferences and residential facility staff preferences.

##### Patient preferences

It is noteworthy that, overall, patient wishes were discussed less than those of families in the included studies. This may be because many of the patients did not have decision-making capacity, due to severe illness or cognitive impairment, or because the studies focussed on the clinician-family relationships. It may also be related to the significant involvement of families in the care of people towards the end of their lives, often including a role in their physical care. That said, one qualitative study found that 76% of hospital admissions in their cohort directly followed patient wishes. Patient needs may also be discussed indirectly and are implicit in other topics such as the adequacy of the current place of care or continuity of care. There was frequent mention of participants’ assessment of a patient’s quality of life, the potential benefits to the patient of a hospital admission and the role of advance care planning all of which are all likely to encompass aspects of the patient’s view.

Advance directives, and advance knowledge of patient wishes, were stated as having high importance by five studies^20,22–25^ and that their presence makes decisions easier. A lack of knowledge and insight into what the patient would have wanted is suggested by some participants as a reason that admission to hospital is more likely. Others described how clinicians considered and felt responsible for the establishing of advance care plans prior to an episode of deterioration as a part of the acute decision-making process.^25^ Given the general drive within health services to improve advance care planning it is somewhat surprising that in many studies the participants did not mention advance care planning at all. This is particularly noteworthy as the majority of the studies were published within the last 10 years.

Twelve of the included studies focussed on patient populations likely to have a high incidence of a lack of capacity (aged residential care residents, those very close to the end-of-life and those with cognitive impairment). Only one actively questions how the clinician makes their assessment of capacity, and how that impacts their decision-making.^26^

A small number of participants referred to the guiding role of clinicians within the decision-making process, helping the patient towards a beneficial decision. They acknowledge that patients are retain in depth medical knowledge of the potential benefits and risks of a hospital admission, and that the clinician is responsible for helping navigate this.^22^

##### Family/caregiver preferences

All the included studies highlight the influence of families, caregivers and surrogates in the clinician’s decision. Overall, families want patients to be admitted and that this has an influence on clinicians. Several participants use language implying this is experienced as a pressure to admit people to hospital, who they would otherwise potentially not,^24–26^ with others describing clinicians as secondary to families in the final decision,^23,27,28^ potentially doing something that they felt not to be in the patient’s best interests.^26^ Conversely there are some participants who speak positively about sharing the decisions with families, and that it is expected that families should be deeply involved.

Some studies engaged in depth with the origins of family pressures. Explanations given are a lack of faith in the ability of the current care setting (generally residential facilities) to look after their relative,^23^ or a lack of faith in the clinician^26^ as well as a view that hospital care is better. One mixed study concluded that family beliefs around better care in hospital played a role in 54% of the admissions they studied.^29^ Clinician participants spoke negatively of this view, and that they felt families underestimated the care setting and community clinicians’ ability to care for people with changing needs.^23^

Another proposed factor impacting families is the difficulty in accepting their loved one is dying^23^; and this may also relate to the clinicians comfort with end-of-life conversations. One participant said: *‘I believed that the patient was dying. . . but after extreme pressure from the patient’s family and with me as a novice physician. . . the patient was placed in an ambulance’* using his role as a novice to explain why he admitted the patient despite thinking they were dying.^21^

Little detail is given on if, or how, clinicians engage with families in cases of disagreement. Where shared decision-making is mentioned^30^ it is unclear how differing views are balanced, and the clinician conceding to family opinion is often described. One study explores the need for collaboration and consensus building between stakeholders, but does not address how this should be managed, acknowledging that at difficult times someone may still panic and the patient will end up in hospital.^22^ Another participant described their role as a navigator within the decision: ‘[*surrogates] direct the destination, and I am the navigator. I. . . will navigate through this medical system to arrive at that destination that they desire*’^25^ accepting that the clinician has a role in guiding patients and families towards the outcome they desire.

Conversely, there are some participants who report that pressure from families to hospitalise may be overemphasised based on cases of conflict being more memorable than those where everyone agreed. It is also likely that there is significant variation between countries in the relative roles of clinicians and families within the decision. For example, in the Belgian studies the authors comment on the role of GPs being less of a gatekeeper of hospital beds than in other countries^27,29,31^ and families having more responsibility for this type of clinical decision.^27^

Overall most content is focussed on the influence families have in encouraging hospital admission. However, there are counter claims to this and it seems likely it is variable depending on different settings and clinical situations.

##### Residential facility staff preferences

Some studies describe the influence of residential facility staff on the clinician’s decision-making; that either that they encouraged admission^24^ or that they preferred residents to stay with them.^25^ Overall, there seems to be considerable variation on the direction of influence residential facility staff have, and the extent. This is likely to relate to the difference in the type of setting where studies were carried out. No clinician participants describe how they weighed up this input against their own views and those of other stakeholders or how they deal with any conflict.

#### Personal experience, views and attributes of the clinician as decision maker

Factors specific to the clinician are often described and will be considered as separate sub- themes: the clinician’s role, knowledge of end-of-life care, intrinsic decision-making factors and risk.

##### The clinician’s role

The specific job roles of the clinician are not considered by any of the included studies, but they are noteworthy as they are associated with different relationships with patients and different areas of expertise. For example, five studies recruited nursing home physicians in Norway and the US.^21,24–26,32^ This role does not exist in some other countries but may imply a high level of familiarity and expertise with frailty and end-of-life care, and it may be relevant to how confident the clinician is in managing hospitalisation decisions. Studies involving general practitioners (GPs) or family practitioners were more likely to describe resource and workload pressures as having a significant influence over their decision-making.^19,21,30,33^

##### Knowledge, confidence and experience with end-of-life care

Many participants specifically describe how their comfort and experience with end-of-life care influences their decision whether to admit someone to hospital.^20,21,23,25,27,34–36^ This suggests that those who are more experienced will be better at identifying the end-of-life and having the confidence to manage patients in the community. Six of these studies were with GPs, who are by definition generalists, and who will have varying interests and experience in end-of-life care.

The difficulty in identifying, with certainty, when someone is approaching the end-of-life is described by participants in four studies.^20,23,34,36^ Two studies focussed on decision-making regarding patients with end-stage heart failure, and both state that identification of the end-of-life in heart failure is particularly difficult and likely to make them more likely to admit to hospital.^20,34^

##### Intuition

Many studies explicitly state, or allude to, the role of difficult-to-articulate ‘gut instinct’ type decision-making factors that are specific to the clinician which was associated with increased GP experience. One participant describes this as ‘*a subconscious consideration of multiple objective factors*’ or ‘*an intuitive feeling*’^30^ They also reported that some clinicians had intrinsic preferences for certain actions, such as keeping older patients at home, although the origins of these intuitions were not explored. Another participant described similar preferences and wishes specifically relating to a serene death at home for a patient at the end-of-life and that different clinician’s dedication to achieving this may vary.^36^

One study considered the ethical principles underlying their participants’ decision-making, finding that beneficence and non-maleficence predominated, even where participants did not explicitly name these concepts.^26^

##### Medicolegal concerns and risk

Three studies, from the UK and Australia, explicitly mention medicolegal concerns impacting on decision-making^28,30,33^ – listing it as one of their key findings. One participant stated that ‘*nobody ever sued a doctor for sending them into hospital*’,^30^ supporting the view that hospitalisation is less risky to the clinician than keeping someone at home. Another group of participants describe clinicians admitting patients to hospital to avoid complaints and medicolegal risk, despite having a viable alternative in the form of a ‘residential in-reach’ service.^28^

#### Clinicians’ interpretation of events and likely outcomes

The way that clinicians interpret the clinical situation and the potential management options are explicitly discussed in nine studies. It may seem an obvious aspect of the decision-making process, but exploring what they might mean by this is important. For example, in one study the authors break down their participants thought processes to: identifying the nature of the acute problem, considering the patient’s underlying condition, the medical options, the likely burden of the admission, the options within the hospital and the timeframe.^19^ A similar breakdown of the clinical facets is seen elsewhere.^30,33^

Consideration of the patient’s quality of life is specifically addressed in only two studies,^24,33^ although this may be more relevant when the patients involved do not have capacity to make the decision for themselves.

Some participants discuss how difficult it is to manage symptoms at the end-of-life and it may lead them to admit to hospital,^23,35^ suggesting that at times hospitalisation may be a central component of good palliative care, if symptoms cannot be managed adequately elsewhere.

#### Adequacy and availability of alternatives, including current setting

Most of the included studies (*n* = 14) placed significance on the clinician’s interpretation of how appropriate the current setting was for the patient (whether home or residential facility), and also what the realistic alternatives to hospital were.

##### Adequacy of the current setting

Considering the adequacy and safety of a patient’s current setting has several facets: the care (whether that is paid or informal/ provided by loved ones), the availability of skilled nursing, the availability of drugs and treatments and the availability of medical cover. This theme also links back concerns from family members about the adequacy of the current setting. The clinician and family may disagree about whether the patient is appropriately cared for where they are, and this must be carefully navigated to avoid further conflict.

Concerns about the adequacy of care and access to nursing are voiced by clinician participants in studies based both in the community and in residential facilities. One survey study found that 85% of hospital admissions in their cohort were influenced by a perceived inadequate care setting.^29^ Participants highlight the importance of patient safety when assessing adequacy of care and some specifically explored in some detail how the care provision can break down and how the clinician might respond to this. Descriptions of how families may have wishes to care for people at home but underestimate at the burden of providing end-of-life care at home, or simply become exhausted.^35^ Other participants felt that that the palliative care expertise of a residential facility is likely to impact on the perceived adequacy of their care.^33^

##### Availability of alternatives

Three studies explore how the availability of alternatives impacts clinicians’ decision-making. This can be seen as a further exploration of adequacy of the current care setting, as remaining in the current setting should always be considered as an alternative to hospital.

Clinician participants within two of these studies looked at patients with end stage heart failure, and specifically describe the inadequacy of alternatives to admission for this group.^20,34^ Another group discuss how the decision is made between hospital and use of a ‘residential in-reach’ service that provides some escalated care to residential home residents. Despite being in a location where this is available and generally well regarded, they describe how some hospital admissions still take place, and that these decisions often involve family pressures, medicolegal concerns and policy adherence.^28^ In short, many of the same reasons reported for admissions where less viable alternatives are available. This suggests that robust admission avoidance schemes may still not negate some of the other factors leading to admission- and more research is needed to consider this further.

##### A perceived lack of choice

Interestingly, many participants describe certain situations where hospitalisation was unavoidable and there being no choice for the clinician to make.^20,28,30,31,34^ Reasons for this ranged from facility policies (*‘head injuries always go to hospital’*^28^), the default medical option in certain cases of deterioration^20,34^ or certain social circumstances being ‘deal-breakers’ and absolutely incompatible with someone staying at home.^30^ Of note, several participants in the heart failure studies stated that clinical deterioration resulted in hospitalisation without any apparent choice for the clinician.^20,34^

#### System factors

Participants frequently describe factors within the healthcare system that influence their decision-making, beyond the availability of alternatives.^19,21,24,30,33,34^ GP workload and time pressure is considered important, with hospital admission at times considered the ‘easier’ option as it transfers responsibility and tasks to the hospital. Some also discuss these factors in relation to alternatives to admission, considering that alternatives are also advantageous if they remove tasks from the GP, while allowing them to maintain a level of responsibility and involvement.

The use of resources is discussed by three studies^24,30,33^ all of whom found that clinicians feel a sense of responsibility to reduce cost associated with secondary care bed usage.

#### Continuity of care

Many clinician participants discuss the importance of continuity of medical care when making decisions, stating that it allows better knowledge of a patients prior wishes. Others also consider the greater likelihood of unfamiliar clinicians (e.g. the out of hours or emergency services) being more likely to admit someone.^19,23,27,29^ This also relates to advance care planning as this is more readily achievable where there is continuity of care, as are conversations with families about a patient’s ongoing health needs. Lack of continuity of care is generally described as a hurdle to decision-making and no suggestions to address this are seen in the included studies. It is discussed by both GPs and nursing home physicians.

## Discussion

### Summary of main findings

This review has identified six main themes describing factors that influence how primary care clinicians approach hospitalisation decisions: navigating the views of other stakeholders, clinician personal factors and experience, clinician interpretation of events and possible outcomes, adequacy of current and alternative care settings, system factors and continuity of care. The terminology and weighting for each theme was different across the studies and not all themes featured in all studies.

Some themes are inter-related to each other either explicitly or implicitly. Clinician personal factors and experience are likely to have an impact on all the other decision-making factors. This is mentioned explicitly by some participants such as the description of how a ‘novice’ physician was more susceptible to the family pressure to admit someone to hospital. The suggestion given by several participants that their role was to guide patients or families through the medical system and help them reach a joint shared decision also implies a level of experience with this type of decision, and it was their responsibility to gain this familiarity and confidence. The way that clinicians interpret the adequacy of current care of the potential alternatives is also likely to be influenced by their experience with end-of-life decisions. Another important consideration for our question is how confident clinicians are at identifying the final year of life, thus triggering them to consider personalised decision-making and avoiding admission, although this question is not adequately addressed by this review. The precise way in which clinicians bring their own experiences and ‘intuition’ to decisions for this group of patients needs more detailed consideration in future, including unpicking how this ‘intuition’ develops and how it may be influenced.

### Comparison to existing literature

It is known that there is significant variation in rates of hospital admission between individual clinicians and that this is exacerbated outside of normal working hours.^11,37^ Work to explore the reasons behind this has suggested a wide array of reasons which seem to vary across settings, without consensus as to the underlying reasons.^11,33,37^ This work therefore spotlights the specific clinical challenge of admissions decisions towards the end-of-life, which encompasses the potential difficulty in proactively identifying the start of the end-of-life phase. A literature review of GP decisions regarding admission for a general population found broadly similar themes (social factors, fear of litigation, GP gender, GP experience, willingness to take risks and GP view of the benefits of the admission)^11^ although they do not include the role of other stakeholders which may reflect a specific element of decision-making in the final year of life. It is a global issue but most of the evidence we have found here is from Europe. This is likely to limit the extent to which it the results are applicable to other healthcare systems.

Our findings relating to the role of patients and families in the decision-making process reflects the general literature on clinical decisions for this group: that patients and families appreciate involvement in their care and ‘shared decision-making’ but that this is conducted with highly variable efficacy and skill.^38–40^ More detailed research would be needed to consider how specifically the views of all stakeholders are balanced within this specific type of decision-making.

### Implications for practice

This review gives some insight into how primary care clinicians approach hospital admission decisions in the final year of life. It suggests that clinicians with more experience and knowledge in identifying and managing the end-of-life are more empowered to make what they feel is the right decision. Clinicians should be encouraged to gain experience and training in identifying the end-of-life and making complex decisions, as well as in communication skills, to facilitate better decision-making which is shared between patients, families and clinicians. Managing complex decisions towards the end-of-life should be seen as a meaningful skill and should be prioritised.

### Strengths and limitations

This review involved the systematic searching for relevant studies and the robust extraction and analysis of relevant data. The narrative description of our findings allows the synthesis of different types of data.

As we predicted some included studies addressed a larger question than we were interested in, meaning that only small amounts of data were relevant. For example, some studies included clinicians from secondary care as well. Study patient populations were also described using generic terminology which did not always make it clear whether these people were likely to be in the final year of life, however all included studies considered their patient population to be at risk of significant harm with unnecessary hospital admission. This also limit the extent to which we can comment on how our themes relate to each other.

There is substantial variation in country and clinical setting, including the role of the clinician, and this is likely to impact on the way they approach decisions.

## Conclusion

This systematic review and narrative synthesis explores what is known about how clinicians approach hospital admissions in the final year of life. It shows a wide breadth of factors, with the views of other stakeholders, especially the patient’s family, and attributes and experiences of the individual clinician, given significant weight.

Further research is needed to explore how clinicians interact with the other stakeholders in a decision, and precisely how the decision is shared and the different views are balanced. This will help us understand if and how stakeholder conflicts are impacting on the quality of decisions. We also need more research into the experiences and values of the clinicians, and how these impact on their decision-making, to inform education and training of future clinical decision makers.

## References

[1] Framework GS . PIG – Proactive Identification Guidance, https://www.goldstandardsframework.org.uk/cd-content/uploads/files/PIG/NEW%20PIG%20-%20%20%2020.1.17%20KT%20vs17.pdf (2016, accessed 17 May 2024).

[2] LafontC GerardS VoisinT , et al Reducing “iatrogenic disability” in the hospitalized frail elderly. J Nutr Health Aging 2011; 15: 645–660.21968859 10.1007/s12603-011-0335-7

[3] Schattner A . The spectrum of hospitalization-associated harm in the elderly. Eur J Intern Med 2023; 115: 29–33. 20230628. 37391309 10.1016/j.ejim.2023.05.025

[4] Cardona-Morrell M Kim JCH Brabrand M , et al What is inappropriate hospital use for elderly people near the end of life? A systematic review. Eur J Intern Med 2017; 42: 39–50. 20170511. 28502866 10.1016/j.ejim.2017.04.014

[5] Booker M Purdy S . Towards new definitions of avoidable hospital admissions. Br J Gen Pract 2022; 72: 464–465.

[6] Cardona-Morrell M Kim J Turner RM , et al Non-beneficial treatments in hospital at the end of life: a systematic review on extent of the problem. Int J Qual Health Care 2016; 28: 456–469. 20160627. 27353273 10.1093/intqhc/mzw060

[7] Etkind SN Lovell N Bone AE , et al The stability of care preferences following acute illness: a mixed methods prospective cohort study of frail older people. BMC Geriatr 2020; 20: 370. 32993526 10.1186/s12877-020-01725-2 PMC7523327

[8] PHE. Research and analysis: older people’s hospital admissions in the last year of life, https://www.gov.uk/government/publications/older-peoples-hospital-admissions-in-the-last-year-of-life/older-peoples-hospital-admissions-in-the-last-year-of-life#main-findings (2020, accessed 10 October 2023).

[9] Gott M Ward S Gardiner C , et al A narrative literature review of the evidence regarding the economic impact of avoidable hospitalizations amongst palliative care patients in the UK. Prog Palliat Care 2013; 19: 291–298.

[10] Simmonds RL Shaw A Purdy S . Factors influencing professional decision making on unplanned hospital admission: a qualitative study. Br J Gen Pract 2012; 62: e750–e756. 10.3399/bjgp12X658278 PMC3481515 23211178

[11] Glette MK. General practitioners decision-making in questions of hospital admissions—a review of the literature. In: European Safety and Reliability Conference ESREL, Portoroz, 2017.

[12] British Geriatrics Society. End of life care in frailty guidance, https://www.bgs.org.uk/ (2020, accessed 12 August 2024).

[13] Kahlon S Pederson J Majumdar SR , et al Association between frailty and 30-day outcomes after discharge from hospital. CMAJ 2015; 187: 799–804. 20150525. 26009583 10.1503/cmaj.150100 PMC4527901

[14] Pialoux T Goyard J Hermet R . When frailty should mean palliative care. J Nurs Educ Pract 2013; 3: 75–84.

[15] Maida V Devlin M . Frailty, thy name is Palliative! CMAJ 2015; 187: 1312. 10.1503/cmaj.1150074 PMC4646755 26574002

[16] Popay J Roberts H Sowden A , et al Guidance on the conduct of narrative synthesis in systematic reviews: a product from the ESRC methods programme. Lancaster: Lancaster University, 2006.

[17] Programme CAS. CASP qualitative studies checklist, https://casp-uk.net/casp-tools-checklists/ (accessed 12 August 2024).

[18] Dixon-Woods M . The problem of appraising qualitative research. Qual Saf Health Care 2004; 13: 223–225. 15175495 10.1136/qshc.2003.008714 PMC1743851

[19] Hazelhoff M Pouw MA Welker GA , et al Factors influencing professional decision making on acute hospital referral in the case of elderly patients with cognitive impairment among general practitioners (GPs) in The Netherlands: a qualitative study. Tijdschr Gerontol Geriatr 2018; 49: 131–138. 29946754 10.1007/s12439-018-0253-9

[20] Glogowska M Simmonds R McLachlan S , et al “Sometimes we can’t fix things”: a qualitative study of health care professionals’ perceptions of end of life care for patients with heart failure. BMC Palliat Care 2016; 15: 3. 26762266 10.1186/s12904-016-0074-y PMC4712523

[21] Glette MK Kringeland T Roise O , et al Exploring physicians’ decision-making in hospital readmission processes – a comparative case study. BMC Health Serv Res 2018; 18: 725. 30231903 10.1186/s12913-018-3538-3 PMC6146774

[22] Forsgarde ES Roost M Elmqvist C , et al Physicians’ experiences and actions in making complex level-of-care decisions during acute situations within older patients’ homes: a critical incident study. BMC Geriatr 2023; 23: 323. 20230524. 10.1186/s12877-023-04037-3 PMC10206590 37226161

[bibr23-02692163241269671] Kouyoumdjian V Perceau-Chambard E Sisoix C , et al Physician’s perception leading to the transfer of a dying nursing home resident to an emergency department: a French qualitative study. Geriatr Gerontol Int 2019; 19: 249–253. 20190108. 30623550 10.1111/ggi.13600

[bibr24-02692163241269671] Cohen-Mansfield J Lipson S . To hospitalize or not to hospitalize? That is the question: an analysis of decision making in the nursing home. Behav Med 2006; 32: 64–70. 16903616 10.3200/BMED.32.2.64-70

[bibr25-02692163241269671] Palan Lopez R Mitchell SL Givens JL . Preventing burdensome transitions of nursing home residents with advanced dementia: it’s more than advance directives. J Palliat Med 2017; 20: 1205–1209. 28504894 10.1089/jpm.2017.0050 PMC5672615

[26] Dreyer A Forde R Nortvedt P . Life-prolonging treatment in nursing homes: how do physicians and nurses describe and justify their own practice? J Med Ethics 2010; 36: 396–400. 20558436 10.1136/jme.2010.036244

[27] Reyniers T Houttekier D Pasman HR , et al The family physician’s perceived role in preventing and guiding hospital admissions at the end of life: a focus group study. Ann Fam Med 2014; 12: 441–446. 25354408 10.1370/afm.1666 PMC4157981

[28] Amadoru S Rayner J-A Joseph R , et al Factors influencing decision-making processes for unwell residents in residential aged care: hospital transfer or Residential InReach referral? Australas J Ageing 2018; 37: E61–E67. 10.1111/ajag.12512 29476607

[29] Reyniers T Deliens L Pasman R , et al Reasons for terminal hospital admissions: results of a survey among family physicians. Palliat Med 2016; 30: NP169–NP170. 10.1177/0269216316659211 27407016

[30] Moore A Croxson C McKelvie S , et al General practitioners’ attitudes and decision making regarding admission for older adults with infection: a UK qualitative interview study. Fam Pract 2019; 36: 493–500. 30219922 10.1093/fampra/cmy083

[31] Reyniers T Houttekier D Cohen J , et al What justifies a hospital admission at the end of life? A focus group study on perspectives of family physicians and nurses. Palliat Med 2014; 28: 941–948. 20140217. 24534726 10.1177/0269216314522317

[32] Cohen-Mansfield J Lipson S . Medical staff’s decision-making process in the nursing home. J Gerontol A Biol Sci Med Sci 2003; 58: 271–278. 12634294 10.1093/gerona/58.3.m271

[33] McDermott C Coppin R Little P , et al Hospital admissions from nursing homes: a qualitative study of GP decision making. Br J Gen Pract 2012; 62: e538–e545. 10.3399/bjgp12X653589 PMC3404331 22867677

[34] Chen JJ Gamble K Graham-Wisener L , et al GP perceptions of the adequacy of community-based care for patients with advanced heart failure in a UK region (NI): a qualitative study. Open Heart 2018; 5: e000734. 10.1136/openhrt-2017-000734 PMC5888440 29632677

[bibr35-02692163241269671] Giezendanner S Bally K Haller DM , et al Reasons for and frequency of end-of-life hospital admissions: general practitioners’ perspective on reducing end-of-life hospital referrals. J Palliat Med 2018; 21: 1122–1130. 20180504. 29727249 10.1089/jpm.2017.0489

[36] Sercu M Renterghem VV Pype P , et al “It is not the fading candle that one expects”: general practitioners’ perspectives on life-preserving versus “letting go” decision-making in end-of-life home care. Scand J Prim Health Care 2015; 33: 233–242. 26654583 10.3109/02813432.2015.1118837 PMC4750732

[37] Calnan M Payne S Kemple T , et al A qualitative study exploring variations in GPs’ out-of-hours referrals to hospital. Br J Gen Pract 2007; 57: 706–713. 17761058 PMC2151785

[38] Brown EL Poltawski L Pitchforth E , et al Shared decision making between older people with multimorbidity and GPs: a qualitative study. Br J Gen Pract 2022; 72: e609–e618. 20220728. 10.3399/BJGP.2021.0529 PMC8999685 35379603

[bibr39-02692163241269671] Bastiaens H Van Royen P Pavlic DR , et al Older people’s preferences for involvement in their own care: a qualitative study in primary health care in 11 European countries. Patient Educ Couns 2007; 68: 33–42. 20070601. 17544239 10.1016/j.pec.2007.03.025

[40] Joseph-Williams N Elwyn G Edwards A . Knowledge is not power for patients: a systematic review and thematic synthesis of patient-reported barriers and facilitators to shared decision making. Patient Educ Couns 2014; 94: 291–309. 20131109. 24305642 10.1016/j.pec.2013.10.031

